# Impact of disease activity on health-related quality of life in systemic lupus erythematosus – a cross-sectional analysis of the Swiss Systemic Lupus Erythematosus Cohort Study (SSCS)

**DOI:** 10.1186/s12865-017-0200-5

**Published:** 2017-03-28

**Authors:** Benjamin Chaigne, Carlo Chizzolini, Thomas Perneger, Marten Trendelenburg, Uyen Huynh-Do, Eric Dayer, Thomas Stoll, Johannes von Kempis, Camillo Ribi, for the Swiss Systemic Lupus Erythematosus Cohort Study Group

**Affiliations:** 1 Department of Internal Medicine Specialties, Clinical Immunology and Allergy, University Hospital and School of Medicine, Geneva, Switzerland; 2 Department of Community Health and Medicine, Clinical Epidemiology, University Hospital and School of Medicine, Geneva, Switzerland; 3 Division of Internal Medicine and Clinical Immunology Lab, Department of Biomedicine, University Hospital Basel, Basel, Switzerland; 4 Nephrology, Hypertension and Clinical Pharmacology, University Hospital Bern, Bern, Switzerland; 5 Clinical Immunology and Allergy, Sion Hospital, Sion, Switzerland; 6 Rheumatology, Kantonsspital Schaffhausen, Schaffhausen, Switzerland; 7 Division of Rheumatology and Immunology, Department of Internal Medicine, Kantonsspital St Gallen, St Gallen, Switzerland; 8 Clinical Immunology and Allergy, University Hospital Lausanne, Rue du Bugnon 46, Lausanne, 1011 Switzerland

**Keywords:** Systemic Lupus Erythematosus, Disease activity, Damage, Health-related quality of life

## Abstract

**Background:**

To assess the impact of disease activity on health-related quality of life (HRQoL) in systemic lupus erythematosus (SLE).

**Methods:**

Cross-sectional study of patients included in the Swiss SLE Cohort Study between April 2007 and June 2014. HRQoL outcomes were based on the Medical Outcome Study Short Form 36 (SF-36). Disease activity was assessed by the SLE Disease Activity Index score with the Safety of Estrogens in SLE National Assessment modification (SELENA-SLEDAI) and by the physican’s global assessment (PGA).

**Results:**

Of the 252 patients included, 207 (82%) were women. Median [interquartile range (IQR)] age was 43 [32–57] years. SLE was active in 125 patients (49.6%). Median [IQR] mental component summary (MCS) in active vs inactive SLE was 40.0 [30.2–51.0] compared to 47.3 [39.2–52.8] (*p* < 0.01) and median [IQR] physical component summary (PCS) 43.7 [37.0–52.8] compared to 49.1 [38.4–55.6], respectively (*p* < 0.05). Increase in SELENA-SLEDAI or increase in PGA were negatively correlated with PCS and/or MCS. After adjusting for gender, age and disease duration, disease activity impacted on both PCS and MCS and all subscales except general health. Active lupus nephritis and musculoskeletal involvement were associated with physical limitations and emotional problems, increased bodily pain and poor social functioning. Low complement and/or presence of anti-dsDNA antibodies were associated with increased fatigue and reduced mental health.

**Conclusions:**

In patients with SLE, HRQoL is reduced in those with active disease. Impact of disease activity on HRQoL dimensions depends on SELENA-SLEDAI system components.

**Electronic supplementary material:**

The online version of this article (doi:10.1186/s12865-017-0200-5) contains supplementary material, which is available to authorized users.

## Background

Systemic lupus erythematosus (SLE) is a chronic autoimmune disease that mainly affects women. This multifactorial disease arises in genetically susceptible individuals upon activation of the innate and adaptive immune system through internal and environmental factors [1]. Clinical presentation is diverse and may include joint, skin, kidney, neurological or hematological involvement [2]. Sustained inflammation in tissues may lead to organ dysfunction and failure. Fatigue and pain are also prominent complaints in SLE patients [3, 4]. SLE activity and damage in addition to fatigue and pain impact on patients quality of life (QoL) [5, 6]. Thus, treatment in SLE should not only aim at decreasing disease activity and damage accrual but also at improving health-related quality of life (HRQOL) [7].

Both lupus-specific QoL questionnaires and the generic Medical Outcomes Study 36-Item Short Form Health Survey (SF-36) have been used to assess HRQOL in SLE [8]. Most SLE studies have used the SF-36, showing that this tool reliably assess HRQOL in this disease [5, 9–11]. SF-36 outcomes have been used as endpoints for treatment efficacy or as prognostic marker [7, 10, 12]. The influence of disease activity on HRQOL is still debated, possibly because only a few studies have examined disease-related organ involvement in relationship with HRQOL [13–19]. Herein we aimed at assessing baseline HRQOL in a cohort of adult SLE patients [2, 20] and to correlate SF36 scores with global disease activity. In order to further emphasize the possible influence of disease activity on SLE, we assessed the impact of organ involvement on HRQOL.

## Methods

### Patients

Cross-sectional data were collected on patients sequentially included in the Swiss SLE Cohort Study (SSCS) between April 2007 and June 2014. Inclusion criteria were: age ≥ 18 years, diagnosis SLE according to the updated ACR classification criteria [21, 22] or the SLICC 2012 criteria [23], completed SF-36 and corresponding data on disease activity, manifestations and treatment. The cohort study was approved by the ethics review boards of all participating institutions and all patients gave written informed consent. Patients included originated from Clinical Immunology, Internal Medicine, Nephrology, and Rheumatology tertiary care centers located both in the French and German-speaking regions of Switzerland.

### Data collection

Data on patient’s age, sex, ethnicity and family history of SLE, dates of first lupus manifestation and diagnosis, clinical and biological characteristics at baseline, disease activity, laboratory parameters, treatment modalities and co-morbidity were collected. HRQoL was assessed by the SF-36 Version 1 [24, 25]. This tool comprises 8 dimension-scales: physical function (PF), role limitations due to physical problems (role physical, RP), bodily pain (BP), general health (GH), vitality (VT), social function (SF), role limitations due to emotional problems (role emotional, RE), and mental health (MH). Each scale ranges from 0 (lowest possible score) to 100 (highest possible score). These 8 dimensions can be summarized into two global scores, the physical component summary (PCS) and the mental component summary (MCS). Expected SF-36 outcomes for an age- and sex-matched population were generated using an algorithm based on the results of a survey conducted on 1200 adults in Western Switzerland [26]. Disease activity was assessed independently of the SF-36 on the same day, using the Systemic Lupus Erythematosus Disease Activity Index (SLEDAI) score with the Safety of Estrogens in Lupus Erythematosus National Assessment (SELENA) modification [27]. Patients were classified in 2 groups according to their SELENA-SLEDAI: inactive SLE (SLEDAI < 4) and active SLE (SLEDAI ≥ 4). Disease activity was also assessed by the Physician’s Global Assessment (PGA) score with a 4–point-Likert-scale, ranging from 0 (inactive disease) to 3 (very active disease). Medication was detailed for disease-modifying drugs (DMARD’s) taken, which were classified in three groups: systemic glucocorticosteroids (GC), anti-malarials (AM) and immunosuppressive agents (IS). All parameters reflected the 4-week period preceding completion of the SF-36 [28].

### Primary outcomes

Primary outcomes were the differences in the eight SF36 dimension scales at baseline in patients with active and inactive disease.

### Statistical analysis

Quantitative variables were expressed as the median ± interquartile range (IQR) and non-parametric statistics were used to analyze the data. *P-values* < 0.05 (two-sided) were considered significant. A linear regression model was used for multivariate analysis, with SF-36 dimension as dependent variables and age, sex, disease duration and SELENA-SLEDAI system components as independent variables. The purpose of this analysis was to identify which of the SELENA-SLEDAI system component were most strongly associated with differences in SF-36 dimensions. Statistical analysis was performed using GraphPad Prism version 6.00 (GraphPad Software, La Jolla, CA), and SPSS Version 22 (IBM Corp Armonk, NY). Spydergrams were generated using Excel Version 14 (Microsoft, Redmond, Washington).

## Results

**Fig. 1:**
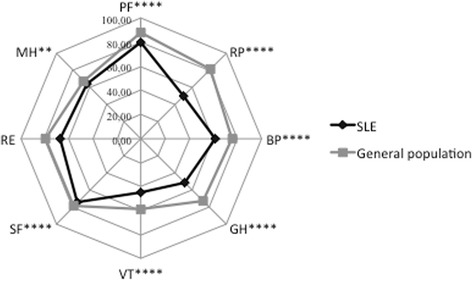
Spydergrams of the eight baseline SF-36 dimensions in 252 patients included in the Swiss Systemic Lupus Erythematosus Cohort Study compared to the expected values in an age- and sex-matched sample of the Swiss general population. PF physical function, RP role physical, BP bodily pain, GH general health, VT vitality, SF social function, RE role emotional, MH mental health. *p < 0.05, **p < 0.01, ***p < 0.001, ****p < 0.0001

Two hundred and fifty-two patients met the inclusion criteria. Patients’ baseline characteristics are shown in Table 1. Approximately half of the patients had inactive disease (Table 1). Those with active disease defined as SELENA-SLEDAI ≥ 4 accordingly had a higher PGA score. They were more often smokers and positive for anti-Sm antibodies. They also had higher ESR values, lower hemoglobin and serum albumin levels and received more often GC and higher average daily prednisone doses (Table 1). HRQoL outcomes in SLE were significantly reduced compared to what is expected in the age- and sex-matched Swiss general population: Median [IQR] PCS in SLE was 46.0 [37.9–54.4] in contrast to expected 52.0 [46.8–57.2], and median [IQR] MCS in SLE was 44.5 [33.6–52.1], in contrast to expected 50.3 [48.7–51.9] (both *p* < 0.001). Except for RE, all other SF-36 dimensions were significantly lower in SLE compared to the expected results in the general population (Fig. 1). Within the SLE group and among general characteristics, only age and body mass index appeared to be negatively correlated with HRQoL outcomes, whereas gender, disease duration and smoking status were not (Table 2). Advanced age negatively impacted mainly on PF and BP, with a median scale of 78.6 and 66.4 in individuals younger than 35 years and 62.3 and 54.5 in those older than 55 years (*p* < 0.001 and *p* = 0.002), respectively. Although to a lesser extent than age, an increase in body mass index also negatively correlated with HRQoL, in particular with PF and BP (Table 2).

**Table 1:** Characteristics of 252 patients with sytemic lupus erythematosus with inactive and active disease at inclusion

Characteristics	All (*N* = 252)	Inactive^a^ (*N* = 127)	Active^a^ (*N* = 125)	*p*-value
Sex, women/men (%)	207/45 (82/18)	104/23 (82/18)	103/22 (82/18)	1.00
Age, median [IQR], years	43 [32–57]	45 [32–59]	42 [32–55]	0.23
Body mass index, median [IQR], kg/m2	24.1 [21.2–27.4]	24.4 [22.0–28.5]	24.0 [20.9–26.7]	0.07
Smoking, no (%)	46 (18)	15 (12)	31 (25)	<0.01
Disease duration, median [IQR], years	6.2 [2.6–14.3]	6.0 [2.9–12.8]	6.6 [2.3–15.1]	0.45
ACR criteria				
Malar rash, no (%)	92 (37)	42 (33)	50 (40)	0.30
Discoid rash, no (%)	46 (18)	28 (22)	18 (14)	0.14
Photosensitivity, no (%)	121 (48)	58 (46)	63 (50)	0.53
Nasopharyngeal ulcers, no (%)	70 (28)	35 (28)	35 (28)	1.00
Arthritis, no (%)	173 (69)	83 (65)	90 (72)	0.28
Pleuritis, no (%)	58 (23)	26 (21)	32 (26)	0.45
Pericarditis, no (%)	44 (18)	23 (18)	21 (17)	0.87
Renal disorder, no (%)	90 (36)	39 (31)	51 (41)	0.12
Seizures, no (%)	12 (5)	4 (3)	8 (6)	0.25
Psychosis, no (%)	13 (5)	6 (5)	7 (6)	0.78
Hematologic disorder, no (%)	155 (62)	73 (58)	82 (66)	0.20
Antinuclear antibodies positive, no (%)	246 (98)	125 (98)	121 (97)	0.44
Anti-Sm antibody positive, no (%)	38 (15)	13 (10)	25 (20)	0.035
Anti-dsDNA antibodies positive, no (%)	150 (60)	68 (54)	82 (66)	0.055
Anti-phospholipid antibodies positive, no (%)	108 (43)	55 (43)	53 (42)	0.90
Laboratory values				
Haemoglobin, median [IQR], g/L	130 [117–137]	131 [122–138]	128 [113–135]	0.011
Leukocytes, median [IQR], G/L	5.9 [4.5–7.7]	5.6 [4.3–7.5]	6.2 [4.6–8.35]	0.12
Platelets, median [IQR], G/L	238 [189–294]	240 [189–285]	236 [185–303]	0.97
Plasma creatinin, median [IQR], μmol/L	70 [61–87]	70 [61–86]	70 [61–92]	0.64
Serum albumin, median [IQR], g/L	39.2 [36.6–42.0]	40.1 [38.8–42.7]	38 [35.0–40.3]	<0.0001
Erythrocyte sedimentation rate, median [IQR], mm/1^st^ hour	12 [6–29]	10 [5–26]	14 [7–34]	0.013
Disease activity and damage				
Physician global assesment, median [IQR]	0 [0–1]	0 [0–1]	1 [0–1.25]	<0.0001
SELENA-SLEDAI, median [IQR]	3 [0.25–8]	1 [0–2]	8 [4–12]	<0.0001
Treatment				
Oral glucocorticosteroids, no (%)	141 (56.0)	55 (43.3)	85 (68)	<0.0001
Daily prednisone equivalent, median [IQR], mg	7.5 [5–12]	5 [5–7.5]	9 [5–20]	<0.0001
Antimalarials, no (%)	178 (71)	91 (71)	87 (70)	0.68
Immunosuppressants, no (%)	120 (48)	55 (43)	65 (52)	0.21

*SD* Standard deviation, *IQR* Interquartile range, *NS* non significant, *SELENA-SLEDAI* Systemic Lupus Erythematosus Disease Activity Index (SLEDAI) score with the Safety of Estrogens in Lupus Erythematosus National Assessment (SELENA), *SDI* Systemic Lupus International Collaborating Clinics/American College of Rheumatology Damage Index

^a^Inactive disease at baseline was defined by a SELENA-SLEDAI < 4 and active disease by a SELENA-SLEDAI ≥ 4

**Table 2:** Spearman’s correlations between baseline characteristics and the eight dimensions and component summaries of the SF-36 in 252 patients with systemic lupus erythematosus

Disease characteristics	PF	RP	BP	GH	VT	SF	RE	MH	PCS	MCS
Age at assessment	−0.27****	−0.08	−0.18**	−0.05	−0.01	0.02	−0.07	0.15*	−0.24***	0.06
Body mass index	−0.25***	−0.12	−0,16*	−0.07	−0.11	−0.13	−0.15*	0.01	−0.18**	−0.05
Disease duration	0.14*	0.13	0.02	0.03	0.11	0.06	0.03	0.14*	0.12	0.02
SELENA-SLEDAI	−0.07	−0.27**	−0.22**	−0.09	−0.15*	−0.14*	−0.27**	−0.16*	−0.14*	−0.21***
PGA	−0.17**	−0.23***	−0.32****	−0.08	−0.13*	−0.13*	−0.19**	−0.16*	−0.21***	−0.14*
ESR	−0.26***	−0.24***	−0.23***	−0.11	−0.13	−0.15*	−0.21**	−0.14*	−0.23***	−0.14*
Haemoglobin	0.16*	0.19**	0.07	0.04	0.05	0.09	0.14**	0.01	0.14*	0.06
Serum albumin level	0.06	0.16*	0.12	0.01	−0.01	0.14	0.11	−0.04	0.12	0.02
Daily prednisone equivalent	−0.03	−0.16	−0.17	0.09	−0.01	−0.18*	−0.10	−0.09	−0.08	−0.10

*ESR* Erythrocyte sedimentation rate, *PGA* Physician’s global assessment, *SELENA-SLEDAI* Systemic Lupus Erythematosus Disease Activity Index (SLEDAI) score with the Safety of Estrogens in Lupus Erythematosus National Assessment (SELENA), *SDI* Systemic Lupus International Collaborating Clinics/American College of Rheumatology Damage Index, *PF* physical function, *RP* role physical, *BP* bodily pain, *GH* general health, *VT* vitality, *SF* social function, *RE* role emotional, *MH* mental health, *PCS* Physical Component Summary, *MCS* Mental Component Summary

*p < 0.05, **p < 0.01, ***p < 0.001, ****p < 0.0001

**Fig. 2:**
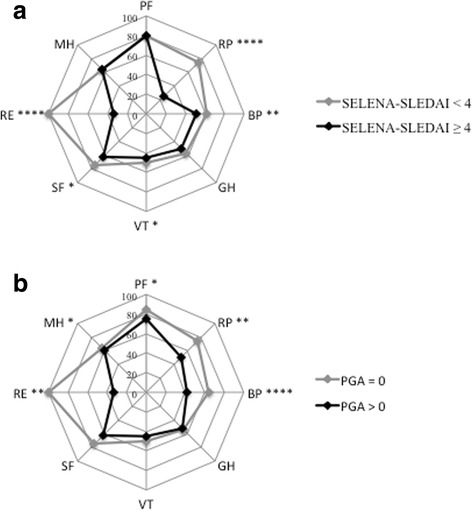
Spydergrams of the eight baseline SF-36 dimensions in 252 patients included in the Swiss Systemic Lupus Erythematosus Cohort Study according to disease activity measures including SELENA-SLEDAI (**a**) and PGA (**b**) scores. SELENA-SLEDAI = Systemic Lupus Erythematosus Disease Activity Index (SLEDAI) score with the Safety of Estrogens in Lupus Erythematosus National Assessment (SELENA); PGA = Physican’s global assessment; PF = physical function; RP = role physical; BP = bodily pain; GH = general health; VT = vitality; SF = social function; RE = role emotional; MH = mental health. *p < 0.05, **p < 0.01, ***p < 0.001, ****p < 0.001

Disease activity had a negative influence on all dimensions of HRQOL, except on GH perception (Fig. 2; Table 2). Accordingly, patients with active SLE had significantly lower MCS (40.0 [30.2–51.0]) and lower PCS (43.7 [37.0–52.8]), compared to patients with inactive disease (MCS 47.3 [39.2–52.8], *p* < 0.01), and PCS 49.1 [38.4–55.6], *p* < 0.05). The association between active SLE and poor HRQoL was confirmed when relying on PGA for disease activity (Fig. 2; Table 2). However, the correlation between BP and disease activity was stronger when assessed by PGA, compared to SELENA-SLEDAI. One the other hand, SELENA-SLEDAI correlated more closely with the RE dimension than PGA. SELENA-SLEDAI correlated more strongly with MCS and PGA with PCS (Table 2).

Among biological activity parameters, an elevated erythrocyte sedimentation rate negatively correlated with most SF-36 dimensions, while hemoglobin and serum albumin levels correlated positively with some (Table 2).

Regarding SLE treatment, GC and IS were associated with active disease, while AM were not (Table 1). Use of GC at the time of visit was associated with a decrease in particular in the RP dimension, with a mean score of 43.8 compared to 66.4 in patients not taking GC (*p* < 0.001).

After adjusting for gender, age and disease duration, disease activity measured by SELENA-SLEDAI impacted on both summary component scales of the SF-36 and all dimension, except GH (Table 3). The effects of the nine SELENA-SLEDAI organ systems on HRQoL are shown in Table 4. (and detailed in Additional file 1: Table S1). Activity in the musculoskeletal, renal and immunological components significantly affected one or more SF-36 dimensions, while there was no significant impact of constitutional signs, serositis, active cutaneous, vascular, and central nervous or hematologic involvement. Musculoskeletal SLE activity defined as arthritis or myositis was strongly associated with BP, but also negatively affected RP, RE and SF, as well as MH. Renal SLE activity defined by hematuria, pyuria, new or increasing proteinuria and/or presence of urinary casts was negatively associated with RE and RP subscales, and to a lesser extent with BP, VT and SF. Immunologic activity defined by low complement levels and/or presence of anti-dsDNA antibodies had a negative impact on VT and MH. Interestingly, the influence of musculoskeletal and renal activity on HRQoL did not depend on age, whereas the impact of vascular involvement became non-significant and that of immunologic activity substantial once adjusted for age, sex and disease duration.

**Table 3:** Linear regression analysis on health-related quality of life outcomes in 252 patients with systemic lupus erythematosus in regard with disease activity age, sex and disease duration

Outcomes	Predictors					
	SELENA-SLEDAI, unadjusted (*N* = 252)			SLEDAI, adjusted for age, sex and disease duration (*N* = 225)		
	Difference in SF-36 score for a 1-point increase in SELENA-SLEDAI	95%-C.I.	*p*	Difference in SF-36 score for a 1-point increase in SELENA-SLEDAI	95%-C.I.	*p*
Physical function	−0.57	(−1.05, −0.10)	0.019	−0.66	(−1.12, −0.19)	0.006
Role physical	−1.86	(−2.63, −1.09)	<0.001	−1.85	(−2.63, −1.07)	<0.001
Bodily pain	−1.16	(−1.68, −0.64)	<0.001	−1.27	(−1.79, −0.74)	<0.001
General health	−0.31	(−0.70, 0.08)	0.12	−0.27	(−0.67, 0.13)	0.19
Vitality	−0.48	(−0.86, −0.10)	0.014	−0.46	(−0.86, −0.07)	0.022
Social function	−0.72	(−1.19, −0.25)	<0.001	−0.71	(−1.20, −0.22)	0.005
Role emotional	−1.92	(−2.73, −1.11)	<0.01	−1.86	(−2.70, −1.01)	<0.001
Mental health	−0.48	(−0.84, −0.13)	0.008	−0.46	(−0.81, −0.11)	0.011
Mental component summary	−0.31	(−0.50, −0.12)	0.002	−0.34	(−0.53, −0.15)	<0.001
Physical component summary	−0.36	(−0.57, −0.15)	0.001	−0.33	(−0.55, −0.11)	0.003

*SELENA-SLEDAI* Systemic Lupus Erythematosus Disease Activity Index (SLEDAI) score with the Safety of Estrogens in Lupus Erythematosus National Assessment (SELENA)

**Table 4:** Impact of disease activity by organ systems assessed through the SELENA-SLEDAI on health-related quality of life outcomes in 252 patients with systemic lupus erythematosus

Predictors	Outcomes															
Unadjusted (*N* = 252)	PF		RP		BP		GH		VT		SF		RE		MH	
SLEDAI organ group	B	p	B	p	B	p	B	p	B	p	B	p	B	P	B	p
Musculoskeletal	−6.75	0.11	−18.45	0.008	−21.89	<0.001	−4.84	0.15	−4.01	0.23	−8.80	0.032	−20.27	0.005	−6.85	0.027
Vascular	−27.12	0.019	−39.45	0.040	−12.96	0.32	−7.51	0.43	−7.13	0.44	−4.79	0.68	−32.58	0.11	1.47	0.86
Renal	−5.35	0.16	−20.68	0.001	−10.07	0.02	−1.87	0.54	−6.88	0.022	−9.99	0.007	−24.41	<0.001	−3.00	0.29
Immunologic	1.88	0.56	−4.96	0.36	2.24	0.54	0.04	0.99	−4.67	0.070	−2.08	0.51	−8.06	0.15	−4.14	0.084
Adjusted^a^ (N = 225)	PF		RP		BP		GH		VT		SF		RE		MH	
SLEDAI organ group	B	P	B	P	B	p	B	P	B	P	B	P	B	P	B	P
Musculoskeletal	−7.40	0.069	−16.29	0.020	−21.85	<0.001	−3.77	0.27	−3.40	0.32	−8.39	0.050	−17.60	0.019	−6.35	0.038
Vascular	−15.29	0.22	−34.71	0.10	−6.98	0.63	−4.56	0.66	−6.24	0.54	−1.76	0.89	−32.29	0.16	−1.75	0.85
Renal	−6.41	0.09	−21.99	0.001	−11.57	0.01	−1.52	0.63	−7.26	0.022	−9.89	0.012	−25.47	<0.001	−2.43	0.40
Immunologic	−1.83	0.59	−6.60	0.26	−0.68	0.86	−0.97	0.73	−6.09	0.030	−3.34	0.35	−8.06	0.19	−5.14	0.041

*B* Regression coefficient: Difference in the SF-36 subscale in patients with organ dysfunction, *PF* physical function, *RP* role physical, *BP* bodily pain, *GH* general health, *VT* vitality, *SF* social function, *RE* role emotional, *MH* mental health, *SELENA-SLEDAI* Systemic Lupus Erythematosus Disease Activity Index (SLEDAI) score with the Safety of Estrogens in Lupus Erythematosus National Assessment (SELENA)

^a^Adjusted for sex, age, disease duration

## Discussion

This study shows a reduction in most HRQoL outcomes in patients with active SLE, when assessed by the SF-36. Global disease activity is a strong predictor of HRQoL, even when adjusted for other factors such as age, sex and disease duration. While nearly all dimension of the SF-36 appear reduced in SLE in contrast to what is expected in an age- and gender-matched general population, with the SLE group active disease defined by a SELENA-SLEDAI ≥ 4 has a dramatic effect on the dimensions that reflect the patient’s role limitations. The relation between global SLE activity and HRQoL was assessed by other cross-sectional as well as longitudinal studies, with conflicting results [13–19]. For instance, two studies by Hanly and Gladman relying on the SF-20 [13, 14] did not find a correlation between disease activity and HRQOL. On the other hand, Stoll et al. using the SF-36 reported a significant association between disease activity assessed by the British Isles Lupus Activity Group System (BILAG) and HRQoL [15].

In this work, we aimed at further deciphering which dimensions were affected by global disease activity and SELENA-SLEDAI system components. Our results show that the SELENA-SLEDAI score with a 4-week window negatively affects every dimension assessed by the SF-36, with the exception of the perceived general health. Disease activity assessed by the physician with a 4-Likert-scale ranging from inactive to very active disease had a similar impact on HRQoL outcomes, with the difference that it was more closely associated with bodily pain and the physical component summary. SELENA-SLEDAI on the other hand was more closely associated with the mental component summary. Overall, global disease activity predominantly affected role physical and role emotional functioning as well as bodily pain. We used the SELENA-SLEDAI organ system classification [27] to differentiate which type of SLE activity impacted most on HRQoL. Active musculoskeletal and renal lupus had a negative influence on most SF-36 dimensions. The impacts of SLE musculoskeletal and renal involvements on HRQoL have been previously reported: In a retrospective study of 303 patients, musculoskeletal flares in the preceding year were independently associated with impairment of most of the subscales of the SF-36, except role limitation due to physical problems and mental health [29]. This is in contrast with our findings, where active musculoskeletal involvement also impaired physical role and mental health, but had no significant effect on physical functioning, general health and vitality. This contrast could be explained by the 4-week window used in our study to assess disease activity, the definition of musculoskeletal involvement based on the SELENA-SLEDAI [30] and by socio-cultural differences in the populations studied. We also found that activity assessed by PGA was more closely correlated with bodily pain than the SELENA-SLEDAI, which emphasizes the importance of the physician’s impression in globally assessing SLE patients. In accordance with previous studies our results underline that controlling musculoskeletal activity is of major importance when aiming at improving HRQoL in SLE.

Only few studies have addressed the impact of lupus nephritis on HRQoL. Vu et al. showed in 1999 that patients with lupus nephritis who progress to end stage renal disease have reduced physical functioning and general health subscales, while mental health appear to improve [31]. Strand et al. reported an improvement of HRQoL in SLE patients treated with sodium abetimus. Strikingly, they also found that the role emotional dimension was significantly reduced in lupus nephritis and that after treatment of renal flares this dimension was also the one to improve the most [32]. More recently, Hanly et al. evaluated the consequence of lupus nephritis on HRQoL. Despite no significant difference in HRQoL outcomes between patients with lupus nephritis and those with non-renal SLE, they showed that patients with advanced renal failure had lower SF-36 subscales (mainly role physical) and summary component scales [33, 34]. Our work shows that among different organs systems assessed for SLE activity, active lupus nephritis had the most pronounced effect on role physical and role emotional functioning. Thus, when assessing response to treatment in patients with lupus nephritis, not only disease activity should be measured but also HRQoL outcomes. Interestingly we found that complement consumption and/or presence of anti-dsDNA antibodies were associated with a decrease in mental health and vitality subscales. The increase in fatigue in immunologically active disease also recalls the results of the BLISS studies, where patients with immunologically active SLE had a better response to belimumab in terms of HRQoL [7]. Altogether these observations may strengthen the impression that immunologic disturbances in SLE are directly responsible for patients’ fatigue and mental alteration. One could hypothesize that pathogenic auto-antibodies, and in particular those targeting dsDNA may exert an effect on the central nervous system [35]. We did not find a correlation between active central nervous system involvement and HRQoL, but this analysis was limited by the fact that only few patients had overt neurologic involvement. Hanly et al. also extensively studied the impact of psychiatric and neurological SLE symptoms on HRQoL. They found that mood disorders and headaches were associated with lower mental and physical component summaries, whereas seizures did not impact on HRQoL outcomes. They however found no correlation between the SLEDAI-2 K score or lupus auto-antibodies and HRQoL [11, 36, 37].

Our study has some limitations. Indeed, most patients in our study population had long-standing SLE. Still our results regarding disease activity are similar to those reported in a recent inception cohort study, where patients with active SLE had poorer HRQOL outcomes [5]. Also, while up to 75% of our patients had been diagnosed with SLE two years or more prior to assessment, HRQoL outcomes in the above mentioned longitudinal study did not change significantly from two years after diagnosis onwards [5]. Thus, we believe that our results regarding disease activity are valid despite the cross-sectional design. We were not able to assess factors such as educational level and presence of fibromyalgia, which are known to negatively impact on HRQoL [18, 38]. Lastly, our study was not able to address the chronicity of SLE, which will need repeated assessment of both disease activity and HRQOL over a longer period.

## Conclusions

In conclusion, our study confirms a low HRQoL in a large cohort of Swiss SLE patients. Disease activity assessed by SELENA-SLEDAI, PGA and ESR all negatively correlate with most HRQoL outcomes. HrQoL was decreased in those with active musculoskeletal and renal involvement and in the presence of classical markers of biological activity.

## Additional file

Additional file 1: Table S1. Impact of disease activity by organ systems assessed through the SELENA-SLEDAI on health-related quality of life outcomes in 252 patients with systemic lupus erythematosus. (DOCX 96 kb)

## Abbreviations

ACR: American College of Rheumatology
BMI: Body mass index
BP: Bodily pain
GH: General health
MCS: Mental components summaries
MH: Mental health
PCS: Physical components summaries
PF: Physical performance
PGA: Physician’s Global Assessment
QoL: Quality of life
RE: Emotional problems
RP: Physical limitations
SCQM: Swiss Clinical Quality Management Program for Rheumatoid Arthritis
SELENA: Safety of Estrogens in Lupus Erythematosus National Assessment
SF: Social functioning
SF-36: Medical Outcome Study 36-item Short Form
SLE: Systemic lupus erythematosus
SLEDAI: SLE Disease Activity Index
SSCS: Swiss SLE Cohort Study
VT: Vitality
