# Supplementary material for: Impact of disease activity on health-related quality of life in systemic lupus erythematosus – a cross-sectional analysis of the Swiss Systemic Lupus Erythematosus Cohort Study (SSCS)
Source: BMC Immunol. 2017 Mar 28;18:17. doi: 10.1186/s12865-017-0200-5 (PMC5371245; doi:10.1186/s12865-017-0200-5)
Supplement: Additional file 1: Table S1. — Impact of disease activity by organ systems assessed through the SELENA-SLEDAI on health-related quality of life outcomes in 252 patients with systemic lupus erythematosus. (DOCX 96 kb) [file 12865_2017_200_MOESM1_ESM.docx]

**Supplementary Table 1.** Impact of disease activity by organ systems assessed through the SELENA-SLEDAI on health-related quality of life outcomes in 252 patients with systemic lupus erythematosus.

| Unadjusted (N=252) | Outcomes; regression coefficient (95%-C.I.); change in SF-36 subscale, if activity in SELENA-SLEDAI organ system present | | | | | | | | | | | |
| --- | --- | --- | --- | --- | --- | --- | --- | --- | --- | --- | --- | --- |
| Predictors | PF | | | RP | | | BP | | | GH | | |
| Constitutional | -13.6 |  | (-31.7, 4.4) | -26.4 |  | (-56.3, 3.6) | -18.7 |  | (-38.9, 1.5) | 9.0 |  | (-5.7, 23.7) |
| Musculoskeletal | -6.7 |  | (-14.9, 1.4) | -18.5 | ** | (-31.9, -5.0) | -21.9 | *** | (-30.7, -13.1) | -4.8 |  | (-11.5, 1.8) |
| Dermal | 0.2 |  | (-6.2, 6.5) | 1.5 |  | (-9.1, 12.1) | -1.7 |  | (-8.9, 5.4) | -0.5 |  | (-5.6, 4.7) |
| Vascular | -27.1 | * | (-49.7, -4.6) | -39.5 | * | (-77.0, -1.9) | -13.0 |  | (-38.5, 12.5) | -7.5 |  | (-26.0, 11.0) |
| Serosal | -3.9 |  | (-17.7, 10.0) | -17.1 |  | (-40.1, 5.8) | -9.4 |  | (-25.0, 6.1) | -7.6 |  | (-18.9, 3.6) |
| CNS | 4.5 |  | (-4.2, 13.2) | -7.3 |  | (-21.8, 7.1) | -4.0 |  | (-13.7, 5.8) | -0.8 |  | (-7.8, 6.3) |
| Hematologic | 3.2 |  | (-5.8, 12.1) | -3.5 |  | (-18.4, 11.4) | -2.9 |  | (-12.9, 7.2) | -0.9 |  | (-8.2, 6.4) |
| Renal | -5.4 |  | (-12.8, 2.1) | -20.7 | *** | (-32.8, -8.5) | -10.1 | * | (-18.3, -1.8) | -1.9 |  | (-7.9, 4.2) |
| Immunologic | 1.9 |  | (-4.5, 8.2) | -5.0 |  | (-15.5, 5.6) | 2.2 |  | (-4.9, 9.4) | 0.0 |  | (-5.1, 5.2) |
|  | | | | | | | | | | | | |
|  | VT | | | SF | | | RE | | | MH | | |
| Constitutional | -4.9 |  | (-19.4, 9.6) | -13.9 |  | (-31.6, 3.9) | -30.4 |  | (-62.0, 1.2) | -11.4 |  | (-24.8, 2.0) |
| Musculoskeletal | -4.0 |  | (-10.6, 2.5) | -8.8 | * | (-16.8, -0.8) | -20.3 | ** | (-34.5, -6.1) | -6.8 | * | (-12.9, -0.8) |
| Dermal | 0.3 |  | (-4.8, 5.3) | -1.3 |  | (-7.5, 5.0) | -3.2 |  | (-14.3, 8.0) | -3.3 |  | (-8.0, 1.5) |
| Vascular | -7.1 |  | (-25.3, 11.0) | -4.8 |  | (-27.2, 17.6) | -32.6 |  | (-72.3, 7.2) | 1.5 |  | (-15.5, 18.4) |
| Serosal | -8.6 |  | (-19.6, 2.4) | -11.4 |  | (-25.0, 2.2) | -14.1 |  | (-38.4, 10.1) | -4.2 |  | (-14.5, 6.1) |
| CNS | 2.4 |  | (-4.5, 9.3) | 1.1 |  | (-7.5, 9.7) | -3.3 |  | (-18.5, 12.0) | -0.8 |  | (-7.2, 5.7) |
| Hematologic | 4.2 |  | (-2.9, 11.4) | -3.3 |  | (-12.1, 5.5) | 4.2 |  | (-11.6, 19.9) | -2.7 |  | (-9.4, 3.9) |
| Renal | -6.9 | * | (-12.8, -1.0) | -10.0 | ** | (-17.2, -2.7) | -24.4 | *** | (-37.1, -11.7) | -3.0 |  | (-8.5, 2.5) |
| Immunologic | -4.7 |  | (-9.7, 0.4) | -2.1 |  | (-8.3, 4.2) | -8.1 |  | (-19.2, 3.1) | -4.1 |  | (-8.8, 0.6) |

| Adjusted for age, sex and disease duration (N=252) | | | | | | | | | | | | |
| --- | --- | --- | --- | --- | --- | --- | --- | --- | --- | --- | --- | --- |
|  | Outcomes | | |  | | |  | | |  | | |
| Predictors | PF | | | RP | | | BP | | | GH | | |
| Constitutional | -17.4 |  | (-35.0, 0.1) | -27.4 |  | (-57.8, 2.9) | -22.5 | * | (-42.8, -2.1) | 10.9 |  | (-3.9, 25.7) |
| Musculoskeletal | -7.4 |  | (-15.4, 0.6) | -16.3 | * | (-30.0, -2.6) | -21.9 | *** | (-30.7, -13.0) | -3.8 |  | (-10.5, 3.0) |
| Dermal | 0.6 |  | (-5.9, 7.1) | 1.8 |  | (-9.4, 13.1) | -2.2 |  | (-9.8, 5.3) | 0.4 |  | (-5.1, 5.9) |
| Vascular | -15.3 |  | (-39.6, 9.1) | -34.7 |  | (-76.6, 7.1) | -7.0 |  | (-35.3, 21.3) | -4.6 |  | (-25.1, 16.0) |
| Serosal | -5.3 |  | (-18.6, 8.0) | -17.2 |  | (-40.0, 5.7) | -9.9 |  | (-25.3, 5.5) | -7.4 |  | (-18.5, 3.8) |
| CNS | 2.9 |  | (-5.5, 11.4) | -7.8 |  | (-22.3, 6.7) | -4.4 |  | (-14.2, 5.4) | -0.2 |  | (-7.3, 6.9) |
| Hematologic | 5.2 |  | (-4.1, 14.6) | -5.5 |  | (-21.7, 10.7) | -1.0 |  | (-11.8, 9.9) | 0.7 |  | (-7.2, 8.6) |
| Renal | -6.4 |  | (-13.8, 1.0) | -22.0 | *** | (-34.5, -9.4) | -11.6 | ** | (-20.1, -3.1) | -1.5 |  | (-7.8, 4.8) |
| Immunologic | -1.8 |  | (-8.5, 4.8) | -6.6 |  | (-18.0, 4.8) | -0.7 |  | (-8.4, 7.0) | -1.0 |  | (-6.5, 4.6) |
|  | | | | | | | | | | | | |
| Organ group | VT | | | SF | | | RE | | | MH | | |
| Constitutional | -3.0 |  | (-17.9, 11.8) | -13.5 |  | (-32.0, 5.1) | -33.5 |  | (-65.9, -1.1) | -8.3 |  | (-21.5, 5.0) |
| Musculoskeletal | -3.4 |  | (-10.1, 3.3) | -8.4 | * | (-16.8, 0.0) | -17.6 | * | (-32.3, -2.9) | -6.3 | * | (-12.3, -0.4) |
| Dermal | 0.8 |  | (-4.7, 6.3) | -2.0 |  | (-8.9, 4.8) | -0.8 |  | (-12.9, 11.2) | -4.3 |  | (-9.1, 0.6) |
| Vascular | -6.2 |  | (-26.7, 14.2) | -1.8 |  | (-27.4, 23.9) | -32.3 |  | (-77.1, 12.6) | -1.7 |  | (-20.1, 16.6) |
| Serosal | -9.0 |  | (-20.1, 2.1) | -11.2 |  | (-25.2, 2.7) | -12.6 |  | (-37.2, 11.9) | -4.6 |  | (-14.6, 5.4) |
| CNS | 2.2 |  | (-4.9, 9.2) | 1.3 |  | (-7.5, 10.2) | -1.6 |  | (-17.2, 13.9) | -1.3 |  | (-7.7, 5.0) |
| Hematologic | 6.8 |  | (-1.1, 14.6) | -3.6 |  | (-13.5, 6.2) | 2.1 |  | (-15.2, 19.4) | 0.3 |  | (-6.7, 7.4) |
| Renal | -7.3 | * | (-13.4, -1.1) | -9.9 | * | (-17.6, -2.2) | -25.5 | *** | (-38.8, -12.1) | -2.4 |  | (-8.0, 3.2) |
| Immunologic | -6.1 | * | (-11.6, -0.6) | -3.3 |  | (-10.3, 3.6) | -8.1 |  | (-20.3, 4.1) | -5.1 | * | (-10.1, -0.2) |

PF = physical function; RP = role physical: BP = bodily pain: GH = general health; VT = vitality; SF = social function: RE = role emotional: MH = mental health; SELENA-SLEDAI = Systemic Lupus Erythematosus Disease Activity Index (SLEDAI) score with the Safety of Estrogens in Lupus Erythematosus National Assessment (SELENA). *** p < 0.001; ** p < 0.01; * p < 0.05.
